# Factors influencing medical students’ attitudes towards substance use during pregnancy

**DOI:** 10.1186/s12909-022-03394-8

**Published:** 2022-05-02

**Authors:** Lou Richelle, Michèle Dramaix-Wilmet, Michel Roland, Nadine Kacenelenbogen

**Affiliations:** 1 Department of General Medicine, Université Libre de Bruxelles, Route de Lennik 808, 612 1070 Brussels, CP Belgium; 2 Department of Epidemiology and Biostatistics, School of Public Health, Université Libre de Bruxelles, Route de Lennik 808, 591 1070 Brussels, CP Belgium

**Keywords:** Substance use, Pregnancy, Medical students, Attitudes

## Abstract

**Background:**

People with substance use disorder, and pregnant women especially, are subject to a lot of stigmas, which can prevent optimal accessibility and quality of care. In this survey, we investigated attitudes of final year medical students regarding substance use during pregnancy and identified the factors that influence these attitudes.

**Method:**

This cross-sectional study was conducted in 2019 and 2020 in Belgium using the short version of the “Substance Abuse Attitude Survey” questionnaire. We focused on two items regarding punishment of substance use during pregnancy. We analysed the concordance between these two, their correlation with other items (e.g. stereotyping, morality, forced withdrawal, low treatment optimism) and the association between respondents’ opinion on punishment and their sociodemographic data.

**Results:**

The response rate was 65.2% (370/567 online and face-to face questionnaires). 19.2% of respondents were in favour of punishment for alcohol use (*n* = 353) and 15.1% for drug use (*n* = 356) during pregnancy. The agreement analysis between the two items showed that 14.3% of students were in favour of punishing both pregnant women who use drugs and those using alcohol. Respondents tended to be more in favour of punishment if they were male students, older, their mothers’ had a lower education level or had no personal or family history of substance use. Attitudes appeared to be more punitive among students with limited contact with people with substance use disorder (i.e. none or limited to hospital). Students intending to specialise in internal medicine were more in favour of punishment of women whereas none of those intending to specialise in psychiatry were in favour.

**Conclusion:**

Our study shows that about 20% of surveyed medical students favoured punishing substance-using pregnant women. Awareness and training work seems to be necessary to ensure adequate care and support for this already vulnerable population.

**Supplementary Information:**

The online version contains supplementary material available at 10.1186/s12909-022-03394-8.

## Introduction

Thirty-five million people worldwide have substance use disorders (SUD) [1]. This is a major and constantly evolving public health concern. Pregnant women are not spared from these disorders and their susceptibility is increasing in certain regions [2, 3]. People who use drugs (PWUD) are stigmatised in numerous and various ways [4, 5], and this is amplified among pregnant women who use substances [6, 7]. This is a complex phenomenon that can be divided into two categories: individual (self) stigma and societal stigma. Self-stigma is defined here as: “the harmful effect that occurs when a person with SUD internalises stereotypes leading to a kind of self-discrimination” [8]. Societal stigmas, on the other hand, are stereotypes, prejudices and discriminations integrated into a community (called social or public stigmas) or an institution (structural stigmas) [9].

Healthcare professionals are not spared from the tendency towards societal stigmatisation of others creating the risk of promoting punitive attitudes rather than support and care for PWUD [10, 11].

Punitive attitudes constitute significant barriers to accessibility and quality of care for these pregnant women with SUD. Indeed, these women are already often on the margins of healthcare systems because of various coexistent vulnerabilities (precariousness, intra-family violence, history of sexual abuse, post-traumatic stress disorder, etc.) and exacerbated stigmas among this population [6, 7]. Pregnant women are not only stigmatised because of the potential risks to the foetus resulting from substance use but also due to perceived gender roles. Since women are socially assigned to the roles of mother and wife, a pregnant woman using drugs violates this social construct and is more likely to face stigmatisation [12].

This stigmatisation increases their risk of marginalisation and exclusion from the healthcare system. That delays these women’s recourse to care for fear of judgment [6, 7, 13] and the socio-legal consequences for them and their progeny such as incarceration, forced treatment, loss of custody of the child etc. [6, 13]. Reduced access to prenatal and postnatal care in women with substance use disorder are demonstrated in the literature [3, 14].

Substance using women of childbearing age, like all women, may or may not desire to have children. If and when they are pregnant, the healthcare system should be able to respond quickly and accompany them adequately, even more because this can be a moment for motivation towards change. Such opportunities should be recognised, supported and followed through [6, 7, 15]. Pregnancy is nevertheless marked by psychological fragility and an increased risk of domestic violence [6, 7, 13].

The question of how medical students and doctors view the punishment of pregnant women has been little explored in the literature. Consequently, the understanding of what can influence such behaviour amongst care providers remains poorly analysed. Assessing the importance of stigma and attitudes among future doctors seems essential towards working on improving the quality of care for this target group. Stigma tends to crystallise and strengthen over time and practice [11, 16], so it is interesting to take stock at a pivotal moment in a future doctors’ practice. Assessment of their behaviour might allow for positive interventions.

In addition, a new law in Belgium seeking to amend the Civil Code with a view to introducing prenatal legal protection (DOC 551029/001) was submitted to the Chamber on 13 February 2020. This law aims to protect the foetus-in-utero of mothers with SUD and/or mental health disorders. The means to protect the foetus could include guardianship, imposed hospitalisation for withdrawal and obligatory caesarean sections.

All this background prompted us to investigate the attitudes of final year medical students (representing future generations of doctors) regarding substance use during pregnancy and analyse what factors influenced their attitudes.

## Methods

We used the short version [17] of the Substance Abuse Attitude Survey (Chappel et al., 1985) [18] a questionnaire validated in the international literature to conduct a cross-sectional study at the Faculty of Medicine of the Université Libre de Bruxelles in 2019 and 2020.

The short questionnaire originally consisted of 25 items. With a committee of experts, we readapted the questionnaire to the Belgian context. We removed questions on marijuana experimentation among young people and on Alcoholics Anonymous, elements less present in Belgium than in the USA. We added a question about paramedical professionals who are much more involved than paraprofessional counselors in our setting. We decided to split the questions on alcohol and drugs in order to be able to assess whether there were different attitudes according to the products consumed. This led to a questionnaire with 29 items (see Additional file 1: Appendix) of good reliability (Cronbach’s Alpha at 0.77). Our considerations were informed by the knowledge that perceptions between what constitutes illegal and legal drugs may differ. Indeed, a general perception of the Belgian population is that the term “drugs” refers de facto to illegal drugs**.** This difference was highlighted in studies among Belgian doctors [19, 20]. In addition, we sought to ensure the transcultural validity of the questionnaire by using bilateral translation and pre-testing the questionnaire amongst doctors with different experiences and amongst lay people as well.

We left the possibility of answering the questionnaire as originally planned, i.e. respondents could position themselves on a 5-point Likert scale (“strongly disagree” to “strongly agree”). We felt it was important to give respondents the option of a “tend to agree/disagree” opinion or to be undecided rather than being forced into a trenchant opinion on contentious items.

In order to assess the socio-demographic dimensions of our participants, we included various questions based on previous studies [21–23]:

1. Socio-demographic type (gender, age, origin)
2. Personal experience (respondent’s personal use or the problematic use by someone in their own social circle/entourage)
3. Orientation towards a particular medical specialty. We did subgroup analyses for the following categories of students:

- Students choosing a specialty directly concerned with the topic of our survey: gynaecology, pediatrics, psychiatry or general medicine
- Internal medicine, which included the largest number of respondents
- Other specialties, including all the remaining specialties

4. Previous personal professional experience (for example encounters with SUD people)
5. Respondent’s personal health (we wanted to assess whether the perception of one’s own health influences the way in which dependent people are perceived). The hypothesis was that a person who considered themselves to be in poorer health would potentially be more empathetic towards patients with this chronic disease. We constructed our question based on the WHO SF-36 quality of life questionnaire [24].

The origin refers here to the nationality of the participants and their parents. By asking about nationalities, we tried to contextualise the cultural environment in which they evolved, which can become a potential determinant of perceptions and attitudes.

The questionnaire was presented to 567 final year medical students in two consecutive years (2019 and 2020). This questionnaire was administered face-to-face to final year medical students in 2019 and online in 2020 given the context of the Sars-Cov-2 crisis.

A total of 370 students responded to the questionnaire with a response rate of 82% in 2019 and 47.3% in 2020 (overall response rate of 65.2%). Thirty two students filled in the questionnaire during registration for optional training on addiction theory and management.

We considered as invalid questionnaires with less than 10 answers, those with answers for only one of the two substances and those where the socio-demographic data were not filled in. Three hundred fifty six completed questionnaires for drugs and 353 for alcohol (350 when the two are crossed) were retained.

For the analysis of the data, we focused on two items in the questionnaire which stated that pregnant women who use substances should be punished with this following formulation: “pregnant women who use drugs (Q18)/alcohol (Q29) should be punished”. The variables were described using frequencies and percentages. The Kappa coefficient was used to measure the agreement between the 2 items. The statistical tests used to compare the proportions were the Chi^2^ and Fisher’s Exact test when the Chi^2^ was not valid. To analyse the correlation between items coded on a 5 option Likert scale, the Spearman non-parametric correlation coefficient (r_s_) was used. STATA SE V16.1 software was used for all analyses and the significance level was set at 5%.

The research protocol was approved by the local ethics committee (Ethics Committee of ERASME-ULB; medical board’s approval number: OM 021) on February 25, 2019, ref.: P2019/156. Informed consent was obtained from all participants who filled out and returned the questionnaire.

## Results

### Overall comment on respondents’ views on punishment

**Fig. 1:**
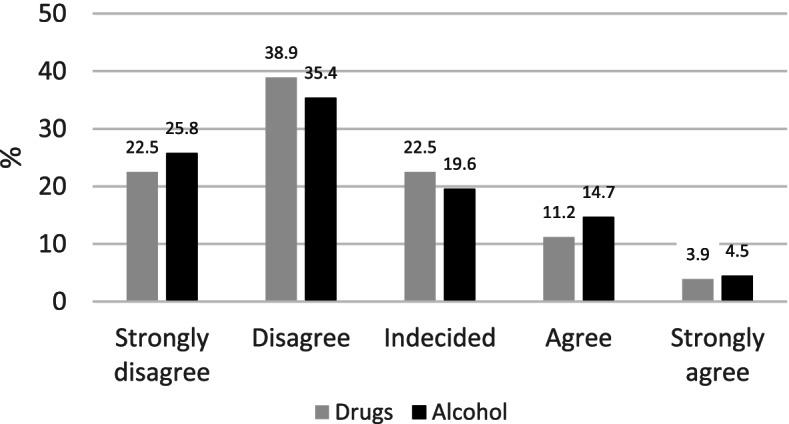
Distribution (%) of responses to questions on punishment if a pregnant woman uses drugs or alcohol.

Of the respondents, 15.1% agreed that women who use drugs should be punished. 19.2% of respondents believed those using alcohol should be punished. In the distribution of responses, the highest percentage response was those who “disagree about punishment”, whether it was for alcohol or drugs. In both cases, there was a relatively large percentage of “undecided” , more pronounced for drugs than for alcohol (22.5% vs. 19.6%) shown in Fig. 1.

The concordance analysis of responses to the two items is shown in Table 1.

**Table 1:** Punishment for pregnant women using drugs or alcohol

	**Alcohol**		
**Drugs**	Disagree *`*(%)	Undecided *n* (%)	Agree *n* (%)
Disagree	**200 (57.1)**	10 (2.9)	6 (1.7)
Undecided	12 (3.4)	**57 (16.3)**	11 (3.1)
Agree	3 (0.9)	1 (0.3)	**50 (14.3)**

% calculated per cell on the total (*n* = 350) - in bold: observed concordance.

Overall, 14.3% of students were in favour of punishing pregnant women both for drug use and for alcohol use. The observed proportion of agreement was 87.7% and the Kappa coefficient, equal to 0.775, showed good agreement.

### Position on punishment and personal characteristics of the respondents

Table 2 shows that certain trends emerged when analysing the associations between the characteristics of the respondents and their responses to items relating to the punishment of pregnant women who use drugs or alcohol. Given the small size of some groups, hardly any of these associations reached statistical significance. A higher percentage of respondents in favour of punishing pregnant women was found among: older people, men, students with mothers with lower levels of education, people who did not use drugs or who did not have substance use disorders in their entourage. There was a statistically significant association between registration or not in optional theoretical training on addiction management (offered by the Department of General Medicine of our faculty) and the opinion on punishment for drug or alcohol use. The percentage of those in favour of punishment was significantly higher among those *not* enrolled in the training.

**Table 2:** Association between respondents’ personal characteristics and agreement on punishment

	Drugs				Alcohol			
	Disagree *n* (%)	Undecided *n* (%)	Agree *n* (%)	*P*^a^	Disagree *n* (%)	Undecided *n* (%)	Agree *n* (%)	*P*^a^
Age (years)				0,378				0,219
< 25	121 (63.0)	41 (21.4)	30 (15.6)		121 (63.0)	36 (18.8)	35 (18.2)	
25–29	86 (63.2)	33 (24.3)	17 (12.5)		81 (60.9)	29 (21.8)	13 (17.3)	
30 et +	10 (50.0)	4 (20.0)	6 (30.0)		9 (45.0)	3 (15.0)	8 (40.0)	
Gender				0,107				0,122
F	155 (65.7)	51 (21.6)	30 (12.7)		151 (64.5)	44 (18.8)	39 (16.7)	
M	63 (55.3)	28 (24.6)	23 (20.2)		61 (54.0)	24 (21.2)	28 (24.8)	
Mother’s education^b^				0,217				0,115
Low	13 (46.4)	7 (25.0)	8 (28.6)		12 (42.9)	8 (28.6)	8 (28.6)	
Middle	39 (63.9)	12 (19.7)	10 (16.4)		39 (65.0)	7 (11.7)	14 (23.3)	
High	162 (63.5)	60 (23.5)	33 (12.9)		157 (62.1)	53 (21.0)	43 (17.0)	
Origin (subjects-parents)^c^				0,843				0,541
Belgium	97 (63.8)	35 (23.0)	20 (13.2)		101 (66.9)	25 (16.6)	25 (16.6)	
Mix BE	31 (57.4)	14 (25.9)	9 (16.7)		31 (58.5)	12 (22.6)	10 (18.9)	
Mix BNE	19 (57.6)	7 (21.2)	7 (21.2)		15 (46.9)	7 (21.9)	10 (31.3)	
Europe	37 (58.7)	14 (22.2)	12 (19.1)		35 (55.6)	13 (20.6)	15 (23.8)	
Outside Europe	29 (70.7)	2 (19.5)	4 (9.8)		26 (63.4)	8 (19.5)	7 (17.1)	
Drug use				0,133				0,231
None	106 (56.7)	47 (25.1)	34 (18.2)		105 (56.5)	39 (21.0)	42 (22.6)	
Cannabis	80 (71.4)	21 (18.8)	11 (9.8)		78 (69.6)	18 (16.1)	16 (14.3)	
Multiple	28 (59.6)	11 (23.4)	8 (17.0)		26 (57.8)	10 (22.2)	9 (20.0)	
SUD in Entourage				0,568				0,464
None	73 (57.9)	28 (22.2)	25 (19.8)		69 (55.7)	25 (20.2)	30 (24.2)	
Alcohol	27 (64.3)	10 (23.8)	5 (11.9)		26 (63.4)	10 (24.4)	5 (12.2)	
Cannabis	11 (57.9)	7 (36.8)	1 (5.3)		9 (50.0)	6 (33.3)	3 (16.7)	
Alcohol-cannabis	43 (70.5)	11 (18.0)	7 (11.5)		42 (67.7)	9 (14.5)	11 (17.7)	
Other drugs	63 (62.4)	23 (22.8)	15 (14.9)		65 (64.4)	18 (17.8)	18 (17.8)	
Health perception				0,475				0,420
Excellent	20 (52.6)	12 (31.6)	6 (15.8)		21 (55.3)	11 (29.0)	6 (15.8)	
Very good	36 (70.6)	9 (17.7)	6 (11.8)		36 (70.6)	8 (15.7)	7 (13.7)	
Good-Fair	19 (63.3)	6 (20.0)	5 (16.7)		17 (56.7)	6 (20.0)	7 (23.3)	
Addiction training subscription				0,028				0,023
No	195 (60.2)	77 (23.8)	52 (16.1)		191 (59.5)	63 (19.6)	67 (20.9)	
Yes	27 (84.3)	3 (9.4)	2 (6.3)		25 (78.1)	6 (18.8)	1 (3.1)	

^a^:Fisher exact or Chi^2^. ^b^:Low = no diploma to lower secondary, Middle = from lower secondary to upper secondary, high = higher education (high school, university, PhD). ^c^: *BE* Belgian and European; *BNE* Belgian and non-European

### Effect of choice of specialty and previous encounters with PWUD on responses

*Higher percentages of students in favour of punishing pregnant women who use drugs were observed for students who wanted to pursue a residency in internal medicine, gynaecology and classified in other specialties (as defined in the methods).*

**Fig. 2:**
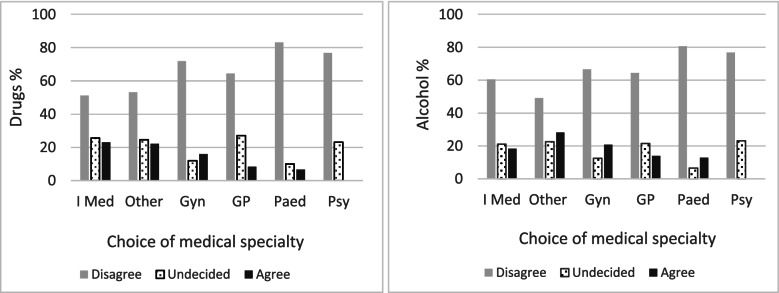
Distribution of responses to the question of punishment for pregnant women who use drugs (figure on the left; *p* = 0.014) or alcohol (figure on the right, *p* = 0.033) according to the medical specialty chosen. Choice of speciality: Internal Medicine (I Med), Other (other specialties), Gynaecology (Gyn), General Practice (GP), Paediatrics (Paed), Child and Adult Psychiatry (Psy)

There were also relatively high percentages of undecided participants for students going into child or adult psychiatry, internal medicine and other specialisations, but also for future general practitioners. The percentage in favour of punishment was nil among psychiatrists (child and adult) followed by future paediatricians. The results were quite similar for alcohol as we can observe in Fig. 2.

**Fig. 3:**
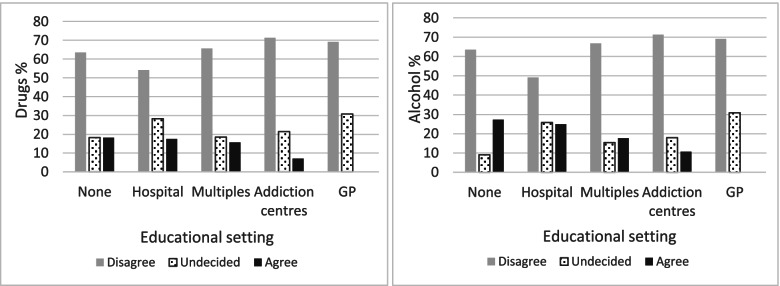
Distribution of responses to the question of punishment for pregnant women who use drugs (figure on the left, *p* = 0.296) or alcohol (figure on the right, *p* = 0.031) according to educational setting. GP = General Practice

Figure 3 shows that the percentage ‘agreeing’ with the punishment of pregnant women using drugs was higher among students who had no contact with people with SUD during internship followed closely by students who had contact in hospital internship only. It was nil among the 13 students who did a traineeship in a GP practice and low among those who had contact in addiction centres. The observations were similar for punishment for alcohol consumption. We were particularly interested in contact with PWUD only in the emergency room, given that this is a special type of contact, as opposed to all hospital contacts, and we did not find any clear difference among those in favour of punishment (18.92% vs.16.87%).

We also looked for correlation between the question/response items relating to stereotypes, morality, forced withdrawal or low treatment optimism and the question/response items about punishment for pregnant women using drugs or alcohol. All the associations, except one, were statistically significant but these associations were weak (r_s_: 0.12 to 0.32). These associations were slightly stronger for alcohol than for drugs. We were also particularly interested in any association between items relating to pregnant women who use drugs and alcohol and the item about coercive pressure for those resistant to trying treatment (which is now included in the law); the three items were categorised as “disagree, undecided, agree”. In both cases the associations were statistically significant (*p* < 0.001) for both drugs and alcohol.

It was observed that 64.8% of respondents in favour of punishing pregnant women who use drugs did not agree with imposed hospitalisation. However, 88.7% of those who were not in favour of punishment did not agree with imposed hospitalisation. Of those who were undecided about punishment 73.8%. were against imposed hospitalisation.

We also note that the percentage of students favouring punishment of pregnant women who use drugs was significantly higher than those in favour of treatment under coercive pressure (15.2% vs. 5.9%), as was the case with the percentage of those undecided about punishment and those in favour of coercive treatment (22.5% vs.12.4% respectively). The results for punishment for alcohol use were quite similar.

## Discussion

Our study highlights some challenging findings, as there is evidence that negative attitudes can be a significant barrier to the care of women who use substances throughout the perinatal period, resulting in delayed care with detrimental health and social consequences for women and newborns [5–7, 10, 13].. The punitive attitudes of the students in our study (up to 20% of which regarding alcohol), although it is not clear what underpins them, seemed less pronounced than in the study by Abel et al. [25]. This study, conducted in the United States in 2002, showed that 52% of the doctors questioned were in favour of passing a law putting drugs or alcohol use during pregnancy in the category of “child abuse” with the aim of removing child custody from these mothers. We are a long way from this result in part to the fact that the setting and the respondents are different but we also know that endorsement of punishment tends to increase with age and clinical practice [11, 16, 23]. However, the literature reports that these attitudes are counterproductive for pregnant women with SUD [2, 26]. Indeed, studies highlight the link between punitive and reporting policies, resulting in fewer pregnant women taking medication-assisted treatment and an increase in neonatal withdrawal syndromes [27]. Punitive policies here are defined as policies by which substance use during pregnancy was criminalised, considered grounds for civil commitment, or considered child abuse or neglect; and reporting policies are defined as policies that mandated reporting of suspected prenatal substance use to relevant authorities.

The fact that the question of punishing pregnant women was asked as one of a series of other general items on PWUD seems to be a strong element of the study, since the responses appeared to be fairly spontaneous and instinctive, hopefully avoiding social desirability bias.

### Characteristics of the respondents

Firstly, our results confirm our hypothesis about the difference in perception between the use of alcohol and other drugs by medical students. In our study, the willingness to punish pregnant women who use alcohol seems more important than for other drugs. We believe that these attitudes are more negative because the students are more familiar with this substance and know more about its effects on women and the newborn baby. Consuming alcohol is part of Belgian culture; Belgian alcohol consumption being higher than the European average, which is already the highest in the world [28]. As in many countries, Belgian university students are particularly engaged in risky alcohol consumption behaviour [29], binge drinking being part of the university tradition. Being familiar with an issue is generally associated with less stigmatisation in studies [22, 23, 30], but in this context, it is a question of considering not only the pregnant women who use drugs and their health, but also the health of the unborn child.

It is also important to note that the use of substances other than alcohol by pregnant women is hardly discussed in the university curriculum, the main focus being on foetal alcohol syndrome. This could partly explain the large number of undecided participants, which is more marked for drugs than for alcohol (23.5% vs. 19.6%).

The punitive approach seems to be influenced by certain characteristics of the respondents. Some, but not all, are already identified in the literature.

Considering that in our sample we are interested in medical students, the majority of our respondents are less than 30 years of age. We can see, however, that there is a tendency to be more punitive towards pregnant women among older respondents. This trend is in line with the literature that shows that stereotypical attitudes increase with age [11, 16, 23].

We also note that gender seems to have an influence on attitudes with more negativity amongst men than women. Studies are divided on the impact of gender on attitudes in relation to PWUD. Attitudes seem to differ according to the substance and familiarity with it [8, 23, 30, 31]. We did not find any studies linking the gender of healthcare professionals and their attitudes in relation to pregnant women with SUD. The fact that in our study, the female gender tends to have a less repressive view could be explained by a phenomenon of identification and projection. Additionally, the target population was young (not yet dealing with parenthood for most of them) and female caregivers are known to be more empathetic than men [32]. It is therefore necessary to be able to qualify other studies concerning attitudes towards PWUD, who are predominantly male [1]. It does not seem surprising to us that male respondents here endorse more punishment towards women using drugs. As mentioned before these women are supposed to be future mothers and to run the household and are commonly deemed to be unfit for maternity [12].

The question of the cultural origin of the students also arises but does not seem to have any influence in our study. The literature is more mixed on this subject [8, 21, 31, 33].

We find no equivalent in the literature to the tendency that students from a less educated environment are more judgmental of people with SUD than those from a higher educated environment (e.g. 28,8% vs 12,9% for drugs). One study highlights less stigma among people with low incomes compared to opiate addiction, which they explain by greater familiarity with opiates in these environments [31].

As regards the choice of future medical specialty, we find few elements of comparison in the literature, since the studies were mainly interested in only certain types of specialisation. There are, however, studies that show that anaesthetists and emergency doctors have the most negative attitudes [10, 11, 23, 34]. Psychiatrists have less of a negative attitude than general practitioners [22]. This is confirmed in our study. Once again, these are attitudes that focused on PWUD and not on pregnant women who use drugs. Unexpectedly, future paediatricians have less repressive attitudes than the future gynaecologists in our study.

Regarding encounters with, or experience of, the SUD problem and in what setting, our sample shows that students who have not been in contact with people with SUD, or who have only been in contact with them in the setting of the hospital, have more punitive attitudes. Those who have been in contact with PWUD in various professional settings, both in the hospital and on an outpatient basis, have less punitive attitudes. It is interesting to note that it is the contact with people with SUD in general practice medicine that seems to have the most favorable impact on the participants’ perceptions, followed by interaction in addiction centres. The fact that patients who consult in these centers are often more precarious, marginalised, and in a poorer overall state of health may perhaps influence medical students. Women with SUD in these centres are often in worse overall health condition than the average patient encountered in general practice. Contacts in the emergency room are often situations where the care relationship can be undermined. The contact is acute, with patients regularly presenting and returning in psychosomatic distress [35]. It seemed important to objectify this point in our study. Unfortunately, as our sample of future emergency physicians was too small (*n* = 11), we could not draw any conclusions. Nor were we able to find any significant difference between contact in the emergency department only versus in the hospital environment in general.

Personal history of substance use or problematic substance use in one’s own entourage seemed to have a rather favourable impact on the attitudes. This is in line with the findings of most studies [10, 19, 23] with rare exceptions [33].

### Possible interventions or actions

We cannot change the socio-demographic characteristics and personal experiences of medical students, but we can work on initial medical training.

Indeed, several studies show that having educational programs and contacts with patients in specialised services can have a positive impact on student development [16, 36, 37]. Studies show that curriculum including repeated interactions with PWUD, preferably in suitable environments, are favourable. Likewise, contacts with pregnant women users of gynaecological services is more favourable where adequate support structures and services have already been set up [16, 38, 39].

Negative attitudes of caregivers towards women consumers are a major obstacle to the quality of care. Such attitudes also have a negative impact on patients’ feelings of empowerment and optimism about treatment and recovery [5, 7]. A less judgmental attitude towards care is therefore beneficial at all levels for women and their newborns.

According to the literature, appropriate care is also efficient at reducing substance use. Opioid-related disorders are considered treatable chronic diseases in pregnant women, especially when they are detected early and receive comprehensive and adequate care [40]. Integrative models accompanying women in a multidisciplinary manner have shown positive health and social benefits for the mother and her newborn [15, 40, 41].

Specialised and multidisciplinary arrangements for these pregnant women consumers exist in Belgium but are still largely insufficient. This fact, as well as a lack of knowledge and positive experiences, may in part explain the high percentage of punishment endorsement.

However, these results should be interpreted with caution. In the questionnaire, we left respondents the possibility of expressing a leaning towards a point of view rather than positioning themselves in a clear-cut manner on contentious issues, which may give rise to debate and invite nuanced answers (cf. odd Likert scale) This option might lead to a large percentage of undecided responses which limits the statistical significance of the results.

Given the sensitive subject and the fact that the questions here were very brief and that the punishment was not clearly defined, it would be interesting to go further and explore this theme in more details by conducting a qualitative study on the topic. It would also be interesting to compare the responses of students starting their medical curriculum with those graduating, as well as responses of more experienced doctors, to be able to assess the influence of the curriculum together with the effects of professional practice on attitudes.

Based on the available data, we can assume that the population of medical students differs from the Belgian population on several levels: women are more represented; there are more people of foreign nationalities and they come from a higher socio-economic background. We cannot exclude that these sociodemographic differences may have impacted the way medical students answered some of the questions in our survey.

## Conclusion

In our study, almost a sixth of students (14.3%) were in favour of punishing both pregnant women who use alcohol and those who use drugs. This result is higher for women using alcohol (19.2%). The scientific literature reports that this attitude is counterproductive and harmful for mothers and unborn children. Our study also notes that certain types of contact with people with substance use disorders reduce these negative attitudes, such as contact in appropriate clinics and contact during an internship with a general practitioner. These positive contacts should therefore be encouraged in the future. It is also important to develop increased awareness of the issue itself, along with the various influences and career attitudes that can arise during training via the university curriculum to promote a vision and ethos of support rather than punishment.

## Supplementary Information

**Additional file 1: Appendix 1.** Questionnaire SAASbis. Socio-demographicdata.

## Data Availability

The dataset used and/or analysed during the current study are available from the corresponding author on reasonable request.

## Abbreviations

SUD: Substance Use Disorder
PWUD: people who use drug
