# Supplementary material for: Factors influencing medical students’ attitudes towards substance use during pregnancy
Source: BMC Med Educ. 2022 May 2;22:335. doi: 10.1186/s12909-022-03394-8 (PMC9060417; doi:10.1186/s12909-022-03394-8)
Supplement: Supplementary file 1 — Additional file 1: Appendix 1. Questionnaire SAASbis. Socio-demographicdata. [file 12909_2022_3394_MOESM1_ESM.docx]

**APPENDIX**

**Appendix 1: Questionnaire SAASbis**

| Indicate your degree of agreement or disagreement by circling the appropriate choice to the right of each statement. There are no right or wrong answers | **Strongly disagree…1** | **Disagree…2** | **Undecided…3** | **Agree…4** | **Strongly agree…5** |
| --- | --- | --- | --- | --- | --- |
| 1. Drug addiction is associated with a weak will | Strongly disagree | Disagree | Undecided | Agree | Strongly agree |
| 2. A drug dependent person cannot be helped until he/she has hit rock bottom | Strongly disagree | Disagree | Undecided | Agree | Strongly agree |
| 3. Heroin is so addicting that no one can really recover once he/she becomes an addict | Strongly disagree | Disagree | Undecided | Agree | Strongly agree |
| 4. Drug abusers should only be treated by specialists in that field | Strongly disagree | Disagree | Undecided | Agree | Strongly agree |
| 5.Smoking leads to marijuana use, which in turn leads to hard drugs | Strongly disagree | Disagree | Undecided | Agree | Strongly agree |
| 6.Daily use of one marijuana cigarette is not necessarily harmful | Strongly disagree | Disagree | Undecided | Agree | Strongly agree |
| 7.Urine drug screening can be an important part of drug abuse treatment | Strongly disagree | Disagree | Undecided | Agree | Strongly agree |
| 8.A physician who has been addicted to narcotics should not be allowed to practice medicine again | Strongly disagree | Disagree | Undecided | Agree | Strongly agree |
| 9.A drug addicted person who has relapsed several times probably cannot be treated | Strongly disagree | Disagree | Undecided | Agree | Strongly agree |
| 10. Long-term outpatient treatment is necessary for the treatment of drug addiction | Strongly disagree | Disagree | Undecided | Agree | Strongly agree |
| 11. Paramedical professionals (psychologists, nurses, social workers,…) can provide effective treatment for drug abusers | Strongly disagree | Disagree | Undecided | Agree | Strongly agree |
| 12.Paraprofessional counselors (trained volunteers, previous drug users) can provide effective treatment for drugs abusers | Strongly disagree | Disagree | Undecided | Agree | Strongly agree |
| 13.Once a person becomes drug-free through treatment, he can never become a social user | Strongly disagree | Disagree | Undecided | Agree | Strongly agree |
| 14.Drug addiction is a treatable illness | Strongly disagree | Disagree | Undecided | Agree | Strongly agree |
| 15.Group therapy is very important in the treatment of drug addiction | Strongly disagree | Disagree | Undecided | Agree | Strongly agree |
| 16.A hospital is the best place to treat a drug addict | Strongly disagree | Disagree | Undecided | Agree | Strongly agree |
| 17.Most drug dependent persons are unpleasant to work with as patients | Strongly disagree | Disagree | Undecided | Agree | Strongly agree |
| 18.Pregnant women who use drugs should be punished | Strongly disagree | Disagree | Undecided | Agree | Strongly agree |
| 19. Coercive pressure, such as threat or punishment, is useful in getting resistant patients to accept treatment | Strongly disagree | Disagree | Undecided | Agree | Strongly agree |

| Indicate your degree of agreement or disagreement by circling the appropriate choice to the right of each statement. There are no right or wrong answers | **Strongly disagree**…**1** | **Disagree…2** | **Undecided…3** | **Agree…4** | **Strongly agree…5** |
| --- | --- | --- | --- | --- | --- |
| 1.Alcoholism is associated with a weak will | Strongly disagree | Disagree | Undecided | Agree | Strongly agree |
| 2.An alcohol or drug dependent person cannot be helped until he/she has hit rock bottom | Strongly disagree | Disagree | Undecided | Agree | Strongly agree |
| 3.Alcohol should only be treated by specialists in that field | Strongly disagree | Disagree | Undecided | Agree | Strongly agree |
| 4. A drug addicted person who has relapsed several times probably cannot be treated | Strongly disagree | Disagree | Undecided | Agree | Strongly agree |
| 5.Alcoholism is a treatable illness | Strongly disagree | Disagree | Undecided | Agree | Strongly agree |
| 6.Group therapy is very important in the treatment of alcoholism | Strongly disagree | Disagree | Undecided | Agree | Strongly agree |
| 7.A hospital is the best place to treat an alcoholic | Strongly disagree | Disagree | Undecided | Agree | Strongly agree |
| 8.Lifelong abstinence is a necessary goal in the treatment of alcoholism | Strongly disagree | Disagree | Undecided | Agree | Strongly agree |
| 9.Most alcohol dependent persons are unpleasant to work with as patients | Strongly disagree | Disagree | Undecided | Agree | Strongly agree |
| 10.Pregnant women who use alcohol or other drugs should be punished | Strongly disagree | Disagree | Undecided | Agree | Strongly agree |

**Socio-demographic data**

What is your gender ?

- - Man
  - Woman
  - Other

1. You are between :
   - 20-24 yrs old
   - 25-29 yrs old
   - 30-34 yrs old
   - 35-39 yrs old
   - >40 yrs old
2. What is your first choice of speciality? *free answer*
3. During your work experiences have you ever been in contact with people with illicit substance use disorder ? (cannabis, cocaine, heroin, amphetamine,…) ? *multiple answers are possible*
   - None
   - Yes, in the emergency room
   - Yes, in a hospital department
   - Yes, during a specialist consultation
   - Yes, in an addiction centre
   - Yes, in a GP consultation
   - Other, please precise: ……
4. Have you ever taken any of these drugs?
   - Yes

- Cannabis
- Cocaine (cocaine, crack)
- Other psychostimulants (amphetamine, methamphetamine, MDMA, mephedrone, …)
- Hallucinogens (LSD, Ketamine, mushrooms, …)
- Hypnotics, sedatives (GHB/GBL, …)
- Opiates (heroin, methadone, buprenorphine, fentanyl, morphine, …)
- Inhalants
- Other, please precise : ______________
  - No

1. Have you ever been in contact within your own environment with one or more problematic use of substances other than tobacco? *several answers are possible*
   - No
   - Yes

- Cannabis
- Cocaine (cocaine, crack)
- Other psychostimulants (amphetamine, methamphetamine, MDMA, mephedrone, …)
- Hallucinogens (LSD, Ketamine, mushrooms, …)
- Hypnotics, sedatives (GHB/GBL, …)
- Opiates (heroin, methadone, buprenorphine, fentanyl, morphine, …)
- Inhalants
- Other, please precise : ______________

1. In general, would you say your health is:
   - Excellent
   - Very Good
   - Good
   - Fair
   - Poor
2. What is the highest level of your parents education?

Father Mother

- - Elementary school a. Elementary school
  - Lower secondary education b. Lower secondary education
  - Higher secondary education c. Higher secondary education
  - High school (High school, University) d. High school (High school, University)
  - None e. None
  - Unknown f. Unknown

1. If you are of foreign origin or if you have opted for Belgian nationality, can you indicate your nationality of origin? …………………………….
2. Are one or both of your parents of a nationality other than Belgian, or have they ever been of a nationality other than Belgian?
   - Yes : ………………………………….
   - No
